# Intracellular Persisting *Staphylococcus aureus* Is the Major Pathogen in Recurrent Tonsillitis

**DOI:** 10.1371/journal.pone.0009452

**Published:** 2010-03-01

**Authors:** Andreas E. Zautner, Merit Krause, Gerhard Stropahl, Silva Holtfreter, Hagen Frickmann, Claudia Maletzki, Bernd Kreikemeyer, Hans Wilhelm Pau, Andreas Podbielski

**Affiliations:** 1 Institute of Medical Microbiology, Virology and Hygiene, Rostock, Germany; 2 Department of Otorhinolaryngology–Head and Neck Surgery, Otto Körner, Rostock, Germany; 3 Institute of Pathology, University Hospital Rostock, Rostock, Germany; 4 Institute of Immunology and Transfusion Medicine, Ernst-Moritz-Arndt University, Greifswald, Germany; Charité-Universitätsmedizin Berlin, Germany

## Abstract

**Background:**

The two major indications for tonsillectomy are recurrent tonsillitis (RT) and peritonsillar abscess (PTA). Unlike PTAs, which are primarily treated surgically, RT is often cured by tonsillectomy only after a series of failed drug therapy attempts. Although the bacteriological background of RT has been studied, the reason for the lack of success of conservative therapeutic approaches is not well understood.

**Methods:**

In a prospective study, tonsil specimens from 130 RT patients and 124 PTA patients were examined for the presence of extra- and intracellular bacteria using antibiotic protection assays. *Staphylococcus aureus* isolates from RT patients were characterized by pulsed-field gel electrophoresis (PFGE), *spa*-typing and MSCRAMM-gene-PCR. Their ability for biofilm formation was tested and their cell invasiveness was confirmed by a flow cytometric invasion assay (FACS), fluorescent *in situ* hybridization (FISH) and immunohistochemistry.

**Findings:**

*S. aureus* was the predominant species (57.7%) in RT patients, whereas *Streptococcus pyogenes* was most prevalent (20.2%) in PTA patients. Three different assays (FACS, FISH, antibiotic protection assay) showed that nearly all RT-associated *S. aureus* strains were located inside tonsillar cells. Correspondingly, the results of the MSCRAMM-gene-PCRs confirmed that 87% of these *S. aureus* isolates were invasive strains and not mere colonizers. Based upon PFGE analyses of genomic DNA and on *spa*-gene typing the vast majority of the *S. aureus* isolates belonged to different clonal lineages.

**Conclusions:**

Our results demonstrate that intracellular residing *S. aureus* is the most common cause of RT and indicate that *S. aureus* uses this location to survive the effects of antibiotics and the host immune response. A German translation of the Abstract is provided as supplementary material (Abstract S1).

## Introduction

Although clearly differing in course of disease, clinical symptoms, and prognosis, RT and PTA have several common aspects: they are comparatively frequent diseases among otolaryngology patients, predominantly or even exclusively caused by bacteria, and despite the possible administration of antibiotics they are successfully managed by surgical measures. Whereas “a chaud” bilateral tonsillectomy and drainage is the method of choice for treating PTA patients [1], [2], RT patients are recommended to undergo surgery when experiencing more than three episodes per year despite adequate antibiotic therapy [3].

Comparably successful treatment regimens for both infections could be due to a similar etiology. In fact, a number of studies have been conducted to elucidate the spectra of bacteria involved in causing PTA or RT. *Haemophilus influenzae*, *Staphylococcus aureus*, and *Streptococcus pyogenes* were - with varying relative proportions - the predominant species isolated from both patient groups (PTA: [4]–[9]; RT: [10]–[26]). Specifically in PTA patients, anaerobes were frequently found to accompany the aforementioned species. While the bacteriological spectra of PTA patient specimens were generally reported without comparison to other patient groups, data from RT patients were compared with data from healthy persons or patients undergoing tonsillectomy because of tonsillar hypertrophy [4], [14], [17]–[19]. Astonishingly little differences were seen between these groups of patients. In RT patients, also the efficiency of different approaches for material collection was compared by employing superficial swabs from the tonsillar surface or the pharyngeal wall vs. fine needle aspirations or surgically prepared tonsillar core [12]–[16], [21], [23], [24], [26]. With the exception of *Haemophilus influenzae* more frequently isolated from the tonsillar core, again little differences could be established between the compared groups. Yet, to our best knowledge a direct comparison of the local microflora in PTA and RT patients utilizing both surface swabs and surgical specimens has not been performed so far.

The reason why at least RT patients often cannot be cured by antibiotic therapy still remains unclear. Low concentrations of the antibiotics in the tonsillar tissue, potentially combined with the presence of resident bacteria producing protective enzymes, or specific antibiotic resistance patterns of the involved pathogenic bacteria have been presented as explanations [27]. In addition, the localization of the causative agents in superficial biofilms or inside the tonsillar tissue could contribute to functional antibiotic resistance in spite of absent specific resistance mechanisms [28]–[32]. While an intracellular localization of *S. pyogenes* in tonsillar cells and an associated resistance to *β*-lactam antibiotics is generally accepted based on few ex vivo and numerous in vitro studies [33], [34], much less is known about the role of intracellular *S. aureus* in upper respiratory tract infections. Generally, *S. aureus* has been demonstrated to internalize with varying efficiency into non-professional human phagocytes [35], [36], but so far was described as an intracellular resident in only few patients with recurrent rhinosinusitis [37], [38].

Invasion of *S. aureus* is influenced by a broad variety of virulence factors, especially adhesins or so called “microbial surface components recognizing adhesive matrix molecules” (MSCRAMMS). Staphylococcal adhesion to host cells is often mediated through binding to bridging matrix molecules, which are likewise bound by the host cells via specific receptors like *β*1-integrins [35]. Certain invasive *S. aureus* strains express for example two fibronectin binding proteins (FnbpA/-B), three proteins for fibrinogen binding: clumping factor A and B (ClfA/-B) and fibrinogen binding protein (Fib) [39], [40]. MSCRAMMS for bone sialoprotein (bone sialoprotein binding protein–Bbp) and collagen (collagen binding protein–Cna) are associated with osteomyelitis and arthritis. Further adhesins, which are common in invasive isolates, are for example elastin binding protein (Ebp) and laminin binding protein (Eno) [39]. Since the most *Staphylococcus* caused diseases are not associated with the expression of single “typical” toxins like toxic shock syndrome toxin, epidermolytic toxins or enterotoxins it was assumed that the combination of a number of factors especially MSCRAMMs during the infective process determines the invasive character of a certain strain [40].

One strategy to evade humoral immunity and the effects of several antibiotics is persistence in the cellular interior. So *S. aureus* has evolved several mechanisms for intracellular persistence. After attaching to host cells via MSCRAMMs *S. aureus* is internalized through clathrin-coated pits following the rearrangement of the cytoskeleton [35]. Staphylococcal invasion is typically associated with the induction of apoptosis and in consequence with the death of tissue (necrosis), but it has also been demonstrated that *S. aureus* has the capability to upregulate cytoprotective or antiapoptotic factors to establish an intracellular carrier state [41]. For example, it was shown that *S. aureus* is able to prevent staurosporine-induced apoptosis by downregulating the cytochrome c release with subsequent caspase-3 activation after its engulfment by macrophages [41]. Some *S. aureus* strains adopted to intracellular environments present an altered metabolism and a reduced production of virulence factors. These strains sometimes grow very slow in small non-pigmented colonies. That's why there were referred to as small colony variants (SCV) [35].

Thus, defining the most prominent bacterial species in RT patients as compared to PTA patients and elucidating the preferred site at which these bacteria persist in between the infection periods could help to develop new strategies for a successful therapy besides the well established but, considering e.g. post-surgical hemorrhages, potentially harmful tonsillectomy [42]. Therefore, swabs and surgically removed specimens from RT and PTA patients and a large panel of complementing methods were used for a prospective and comprehensive study of the involved bacterial species and their precise anatomical location.

Here, we show that *S. aureus* is the predominant species in RT patients. According to our results, long term persistence is likely due to an intracellular location rather than biofilm formation. This feature was not associated with a single, especially successful clone but with a wide variety of strains even when looking at a locally restricted group of patients. The antibiotic resistance patterns of the RT-associated isolates did not significantly differ from other outpatient *S. aureus* isolates and thus, did not offer a straightforward conservative approach to eradicate the bacteria from their intracellular asylum.

## Results

### Patient Data

Age and gender ratio of the 130 RT patients and the 124 PTA patients are listed in table 1. The mean age of PTA patients was 11 years higher than in the RT group. Similarly, the median age of children among the PTA patients was 16 years as opposed to 8.5 years among the RT patients. These differences were highly significant. Thus, PTA occurred more frequently in adolescents and adults whereas RT was more frequent in children and younger adults. According to our data female patients predominated in the RT group, whereas the gender ratio was more balanced in the PTA group (table 1).

**Table 1:** General caracteristics of the study population.

		RT			PTA		
Characteristics	Childrenn = 44	Adults n = 86	All n = 130	Children n = 21	Adults n = 103	All n = 124	p-value All/All
Age (mean) yrs	8.8±5.0	29.1±10.0	22.3±12.9	13.7±3.6	36.9±15.8	33.0±16.9	0.0000
Age (median) yrs	8.5±4.5	27.0±8.2	20.0±10.4	16.0±2.8	34.0±13.1	28.0±13.9	
Male sex n (%)	14 (31.8)	34 (39.5)	48 (36.9)	12 (57.1)	58 (56.3)	70 (56.5)	0,0025^*^

•according to Wilcoxon-Mann-Whitney-U-Test.

All RT patients showed the typical clinical signs of tonsillar hypertrophy (grade: 1: 9.2%; 2: 50.0%; 3: 40.8%) and tonsillar fixation (grade 1: 30.3%; 2: 57.9%; 3: 11.8%). Clefted tonsils, cervical lymphadenopathia and tonsillar exsudate were quite common symptoms among the *S. aureus* RT patients. In contrast, abcess formation and tonsillar erythema were remarkably rare among them (table 2).

**Table 2:** Anamnestic data of the RT-patients.

patient	Hypertrophy grade	Tonsillar fixation	Tonsillar erythemia	Clefted tonsils	abscess	cervival lymph-adenopathy	pus/exsudate detritus
G64I	2	1	−	+	−	−	+
A56	3	1	−	+	−	−	+
A54	3	1	−	+	−	−	+
G64II	2	1	−	+	−	−	+
A57	2	2	−	−	−	+	−
G57	3	1	−	+	−	+	−
A55	2	1	−	+	−	−	+
A13	1	2	−	+	−	−	−
A22	3	3	−	+	−	+	−
G72	3	1	−	+	−	+	−
G88	3	2	−	+	−	+	+
A41	2	3	−	+	−	+	−
G32	2	1	−	+	−	+	+
G20	2	2	−	+	−	+	−
A05	2	2	−	+	−	+	−
A34	2	2	−	+	−	−	+
A20	1	2	−	+	−	−	−
G71	3	1	−	+	−	+	+
G46	2	1	−	+	−	−	−
G47	2	1	−	+	−	−	−
G36	2	2	−	+	−	−	+
G53	3	2	−	+	−	−	−
G61	2	2	−	+	−	−	−
A07	3	2	−	−	−	+	−
G80	3	1	−	+	−	+	−
G90	3	2	−	+	−	+	+
G43	2	2	−	+	−	−	−
G91	2	2	−	+	−	−	+
A27	3	3	−	+	−	+	+
G14	2	2	−	+	−	−	−
A03	2	2	−	+	−	−	+
A15	1	2	−	−	+	−	−
G29	2	2	−	+	−	+	+
A36	1	2	−	+	−	−	−
G12	3	2	−	+	−	+	−
G11	2	1	−	+	−	+	−
G07	3	2	−	+	−	−	−
G21	2	2	−	+	−	−	−
G30	3	1	−	+	−	+	+
G09	2	2	−	+	−	+	+
G51	3	2	−	+	−	−	+
A35	2	2	−	+	−	−	−
G49	3	1	−	+	−	+	−
A14	3	2	−	−	−	−	−
G10	1	1	−	+	−	−	+
G77	3	2	−	+	−	−	−
G75	3	1	−	+	−	+	−
G45	3	2	−	+	−	−	+
G62	2	3	−	+	−	−	−
G50	3	2	−	+	−	−	+
G76	2	2	−	+	−	−	+
G27	3	1	−	+	−	−	−
G41	3	2	−	+	−	−	+
A37	2	2	−	+	−	+	−
G22	2	3	+	−	−	+	−
G06	2	2	−	+	−	+	−
A12	3	1	−	−	−	−	−
G31	2	2	−	+	−	+	+
G78	2	2	−	+	−	+	−
A26^1^	2	3	−	+	+	−	+
G19	2	2	−	+	−	−	−
A02	3	2	−	+	−	+	+
A04	1	3	−	+	−	−	+
A43	3	1	−	+	−	+	+
A33	3	3	−	+	−	+	+
G04	2	2	−	+	−	+	−
G55	1	1	−	+	−	−	−
G56	2	2	−	+	−	−	+
A42	3	2	−	+	−	+	−
G86^2^	2	2	−	+	−	+	−
G84	2	2	−	+	−	−	−
A44	3	1	−	+	−	+	+
A39	2	3	−	+	−	+	−
A17	2	2	−	+	−	−	+
G35	2	1	−	+	−	+	+
G87	3	2	−	+	−	−	−
% positive	100	100	1.3	92.1	2.6	46.1	43.4

Signs and symptoms were graded as “0”–none, “1”–poor, “2”–moderate. “3”–strong).

### Detection of Bacterial Pathogens

Table 3 lists the results of the culture techniques applied on the tonsillar material prior to exposure to antibiotics. *Neisseria* sp., *α*-hemolytic streptococci and coagulase-negative staphylococci as typical members of the resident microflora in the upper respiratory tract are not listed in this table. Notably, there were 31 PTA patients (25.8%) but only one RT patient (0.7%), which exclusively presented typical microflora of the upper respiratory tract.

**Table 3:** Distribution of potentially pathogenic microorganisms isolated from RT and PTA patients.

		RT			PTA		p-value
Bacterial species	Children n = 44	Adults n = 86	All n = 130	Children n = 21	Adults n = 103	All n = 124	RT/PTA All/All
*Staphylococcus aureus * MRSA^a^ SCV^b^	29 (65.9) 0 (0) 0 (0)	46 (53.5) 1 (1.2) 1 (1.2)	75 (57.7) 1 (0.8) 1 (0.8)	4 (19.0) 0 (0) 0 (0)	6 (5.8) 0 (0) 0 (0)	10 (8.1) 0 (0) 0 (0)	0.0000 1.0000 1.0000
*Streptococcus agalactiae*	3 (6.8)	4 (4.7)	7 (5.4)	0 (0)	3 (2.9)	3 (2.4)	0.3353
*Streptococcus anginous*	4 (9.1)	18 (20.9)	22 (16.9)	1 (4.8)	9 (8.7)	10 (8.1)	0.3810
*Streptococcus constellatus*	7 (15.9)	25 (29.1)	32 (24.6)	3 (14.3)	19 (18.5)	22 (17.7)	0.2200
*Streptococcus dysgalactiae ssp. equisimilis*	1 (2.3)	3 (3.5)	4 (3.1)	1 (4.8)	1 (1.0)	2 (1.6)	0.6843
*Streptococcus pneumoniae*	1 (2.3)	0 (0)	1 (0.8)	0 (0)	2 (1.9)	2 (1.6)	0.6148
*Streptococcus pyogenes*	7 (15.9)	9 (10.5)	16 (12.3)	7 (33.3)	18 (17.5)	25 (20.2)	0.1240
*Haemophilus influenzae*	14 (31.8)	16 (18.6)	30 (23.1)	0 (0)	2 (1.9)	2 (1.6)	0.0000
*Haemophilus parainfluenzae*	9 (20.5)	33 (38.4)	42 (32.3)	1 (4.8)	1 (1.0)	2 (1.6)	0.0000
*Eikenella sp.*	0 (0)	0 (0)	0 (0)	1 (4.8)	1 (1.0)	2 (1.6)	0.2373
*Capnocytophaga sp*	1 (2.3)	1 (1.2)	2 (1.5)	0 (0)	4 (3.9)	4 (3.2)	0.4378
*Enterobacteria*	0 (0)	1 (1.2)	1 (0.8)	0 (0)	2 (1.9)	2 (1.6)	0.6148
*Burkholderia cenocepacia*	2 (4.5)	1 (1.2)	3 (2.3)	0 (0)	0 (0)	0 (0)	0.2475
Anaerobes^c^	-d	-d	-d	8 (38.1)	30 (29.1)	38 (30.6)	-
*Candida sp.*	8 (18.2)	11 (12.8)	19 (14.6)	1 (4.8)	5 (4.9)	6 (4.8)	0.0108

a *Methicillin-resistant Staphylococcus aureus*: Oxacillin MIC ≥4 µg/ml.

b small colony variant.

c predominantly *Prevotella sp.*, *Bacteroides sp.* or *Fusobacterium sp.*

d methods for detection not included in the study protocol.

In the RT group, the most prevalent pathogenic bacterial species was *S. aureus* (75 patients; 57.7%; P<0.001), whereas in the PTA group *S. pyogenes* had this status (25 patients; 20.2%; P = 0.12). Remarkably, *Haemophilus influenzae* (23.1% vs. 1.6%; P<0.001), *Haemophilus parainfluenzae* (32.3% vs. 1.6%; P<0.001) and *Candida* sp. (14.6% vs. 4.8%; P = 0.0108) occurred predominantly in the RT group. The same differences and significance levels were seen when comparing children and adults between both patients groups. Consistently, when matching the results from children and adults within each patient group, no significant differences were apparent, with the exception of *H. parainfluenzae* which was preferentially isolated from adults of the RT group (p = 0.0479).

Table 4 details the microorganisms co-isolated from the 75 *S. aureus* positive RT-patients. In about one fifth of such patients (13 persons) *S. aureus* was the only pathogenic agent. Besides *S. anginosus* and *S. constellatus*, *H. parainfluenzae* and *H. influenzae* predominated among the potentially pathogenic bacterial species accompanying *S. aureus*, while *S. pyogenes* and *S. aureus* co-existed in only one patient. Again, there were no significant differences between the age groups among the RT patients.

**Table 4:** List of potentially pathogenic microorganisms co-isolated with *S. aureus* in RT patients.

	RT n (%)			
Bacterial species	Children n = 29	Adults n = 46	p-value Childen/Adults	All n = 75
only *Staphylococcus aureus* ^a^	5 (17.2)	8 (17.4)	1.0000	13 (17.3)
second *Staphylococcus aureus*	0 (0)	1 (2.2)	1.0000	1 (1.3)
*Streptococcus agalactiae*	3 (10.3)	2 (4.3)	0.3687	5 (6.7)
*Streptococcus anginous*	4 (13.8)	12 (26.1)	0,2561	16 (21.3)
*Streptococcus constellatus*	3 (10.3)	9 (19.6)	0,3491	12 (16.0)
*Streptococcus dysgalactiae ssp. equisimilis*	0 (0)	1 (2.2)	1.0000	1 (1.3)
*Streptococcus pneumoniae*	1 (3.4)	0 (0)	0.3867	1 (1.3)
*Streptococcus pyogenes*	2 (6.9)	1 (2.2)	0.5555	3 (4.0)
*Haemophilus influenzae*	9 (31.0)	6 (13.0)	0,0774	15 (20.0)
*Haemophilus parainfluenzae*	7 (24.1)	21 (45.7)	0.0864	28 (37.3)
*Capnocytophaga sp.*	1 (3.4)	2 (4.3)	1.0000	3 (4.0)
*Candida sp.*	6 (20.7)	4 (8.7)	0.1715	10 (13.3)

In addition to the listed species, in all patients bacteria of the physiological resident microflora could be detected.

**Figure 1:**
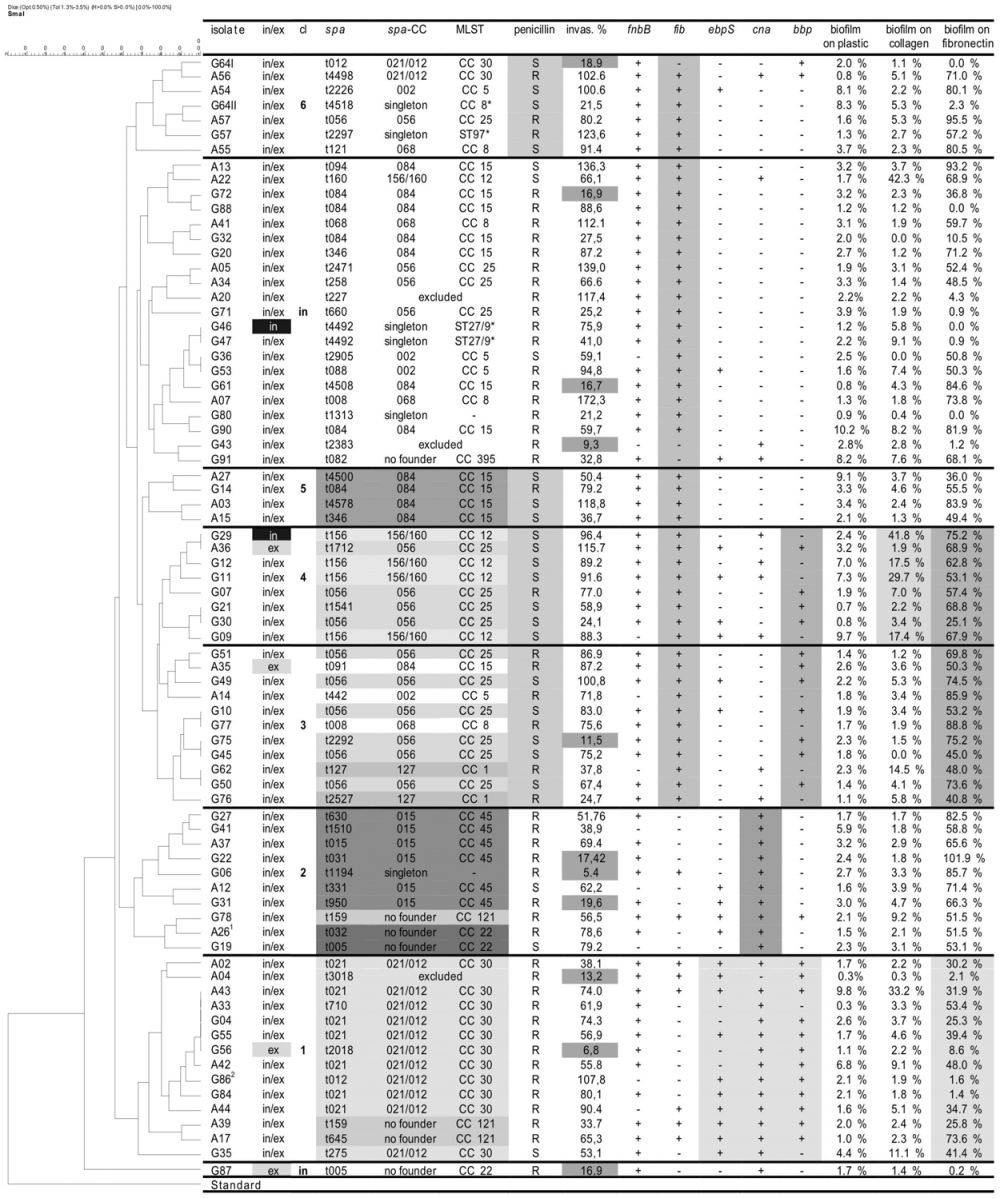
UPGMA-tree of PFGE patterns and group-associated characteristics of *S. aureus* isolates from RT patients. The consensus tree was constructed by interpreting PFGE-band patterns with help of the GelCompar™-Software (version 4.1, Applied Math) using the UPGMA algorithm with Dice coefficients. Based on this consensus tree and taking the screened parameters listed in this table into account 6 clusters and a group of “independent” or unrelated strains can be distinguished; Legend: in/ex–intracellular/extracellular location according to the antibiotic protection assay, dark/grey fields denote the exclusive presence at the intra-/ extracellular location; cl–cluster number, in–independent strain, *spa*–*spa*-type; *spa* CC–*spa*-clonal cluster; MLST–Multi-Locus-Sequence-Typing clonal clusters, MLST-CCs were deduced from BURP grouping of *spa*-typing; ST–Sequence Type; penicillin–penicillin susceptibility (S-susceptible, R-resistant); invas. %–invasive character of the isolate in the FACS-based invasion assay, cut off: <20% value relative to the corresponding value of *S. aureus* strain Cowan I during the same series of experiments, grey fields denote non-invasive strains; *fnbB*–fibronectin binding protein B; *fib*–fibrinogen binding protein; *ebpS*–elastin binding protein; *cna*–collagen binding protein; *bbp*–bone sialoprotein binding protein, (note: 100% of the isolates were positive for clumping factor A & B (*clfA*, *clfB*) fibronectin binding protein A (*fnbA*) and laminin binding protein (*eno*)), biofilm formation of the RT-associated *S. aureus* isolates in relation to *S. aureus* strain ATCC 25923; (The different gray shaded boxes indicate groups of isolates with above average frequency of the certain parameter) ^1^ MRSA; ^2^ small colony variant.

*S. aureus* was most frequently detected in both extracellular and intracellular reservoirs in the tonsils (figure 1). However, in tonsils of 4 RT patients *S. aureus* was cultured exclusively extracellular, i.e., before in vitro exposure to antibiotics. In contrast, tonsil material of only two RT patients appeared to be sterile, but *S. aureus* was cultured from the intracellular compartment with the antibiotic protection assay, indicating an exclusively intracellular localization of the bacteria.

One *S. aureus* isolate (G86) grew in tiny, nonpigmented, nonhemolytic colonies typical for a small colony variant but reverted immediately after one passage to the normal morphotype.

#### Susceptibility testing data

Table 5 compares the antibiotic susceptibility patterns of the 76 *S. aureus* isolates from the RT patients with *S. aureus* isolates of 296 outpatients from the Health Centers of the departments of otolaryngology, ophthalmology and oral and maxillofacial surgery of the University Hospital Rostock between 2001 and 2007. In general, there were no significant differences for all the tested antibiotics.

**Table 5:** Antimicrobial susceptibility of *S. aureus* isolates from RT patients and reference strains from different upper respiratory tract-related outpatient clinics^1^ of the Rostock University Hospital in the years 2001–2007.

	n (%) susceptible *S. aureus* isolates		
antibiotics	*S. aureus* strains of outpatients with RT n = 76	*S. aureus* strains of patients from outpatient clinics^1^ n = 296	p-value
penicillin	25 (32.9)	70 (23.6)	0.1063
Oxacillin	75 (98.7)	296 (100)	0.2043
cefuroxime	75 (98.7)	296 (100)	0.2043
levofloxacin	75 (98.7)	285 (96.3)	0.4724
moxifloxacin	75 (98.7)	-	-
gentamicin	75 (98.7)	285 (96.3)	0.4724
streptomycin	66 (86.8)	-	-
erythromycin	70 (92.1)	271 (91.6)	1.000
teicoplanin	76 (100)	296 (100)	1.000
vancomycin	76 (100)	296 (100)	1.000
cotrimoxacol	76 (100)	293 (99.0)	1.000
doxycycline	71 (93.4)	260 (87.8)	0.2181
clindamycin	76 (100)	284 (95.9)	0.1366

1 The material was collected from outpatients presenting at the otolaryngology, ophthalmology, and oral and maxillofacial surgery clinics.

Only from one 29 year old female RT patient a methicillin-resistant *S. aureus* strain was isolated. All other strains were susceptible to oxacillin, cefuroxime, levofloxacin, moxifloxacin and gentamycin. All strains including the MRSA-isolate were susceptible to vancomycin, teicoplanin, cotrimoxacol and clindamycin.

#### FACS-based eukaryotic cell internalization

**Figure 2:**
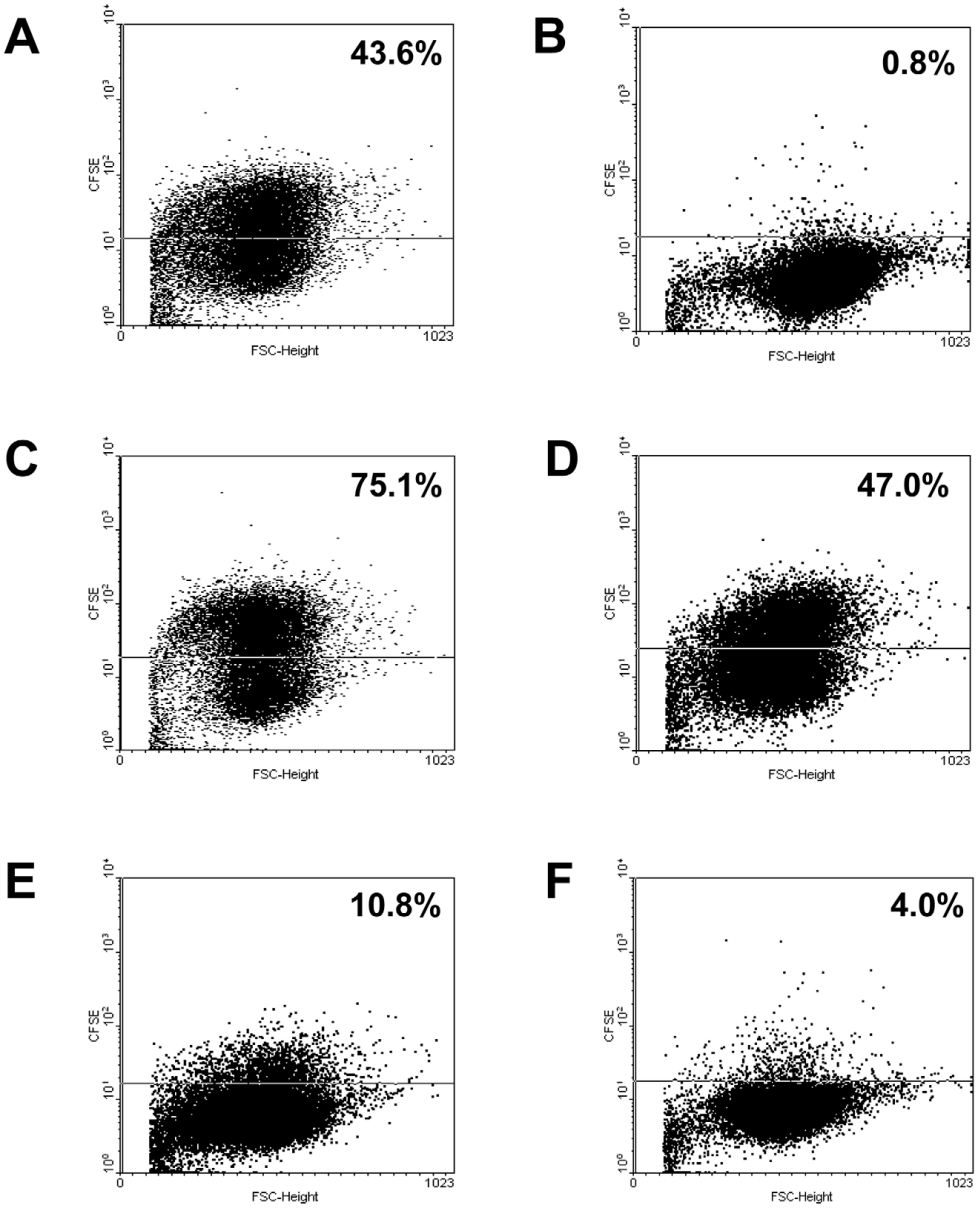
*S. aureus* invasion of human A549 lung carcinoma cells. Shown are the original dot plots of representative flow cytometric analyses of CFSE-labeled bacteria after addition of 0.2% trypan blue. The percentage of cells containing intracellular staphylococci is written in the upper right corner of the plot. (A) *S. aureus* strain Cowan I–positive control strain with good invasion (100%); (B) *S. carnosus*–negative control strain showing no invasion, The percentage of cells containing intracellular *S. carnosus* is subtracted from that of all other *Staphylococcus* isolates including the Cowan I control (0%); (C) *S. aureus* isolate A7–highly invasive strain (172.3% of Cowan I); (D) *S. aureus* isolate G86–cell internalization rate comparable to the positive control strain (107.8% of Cowan I); (E) *S. aureus* isolate G76–very low invasive strain (24.7% of Cowan I); (F) *S. aureus* isolate G43–non-invasive strain (9.2% of Cowan I, below 20%).

The results of the FACS-based internalization assays are presented in figure 1 and 2 presents a selection of representative original dot plots. The range of non-invaded and invaded cells is defined by a fluorescence of less than 1% and 55–75% of eukaryotic cells. The contribution of extracellular adhering bacteria to the fluorescence signal was found to be generally lower than 6% of the tested cells. Setting the results of the positive control as 100% and defining 20% of this value as the cut-off mark for non-invasiveness, 85.5% (65/76) of the *S. aureus* strains eagerly invaded A549 cells. The average relative internalization ratio among these strains was 74% (±31%). Since 57.7% (75/130) of all RT patients were positive for *S. aureus*, 50% (65/130) of all RT cases within the study population were associated with invasive *S. aureus* isolates.

#### Biofilm formation

As shown in figure 1, none of the tonsillar *S. aureus* isolates grew monospecies biofilms on uncoated polystyrene surfaces, whereas the reference strain *S. aureus* ATCC 25923 was an excellent biofilm former in this experimental setup.

After coating the polystyrene surfaces with human collagen, only 4 (5.2%) of the 76 strains demonstrated a moderate to good (>20%) biofilm formation capability. In contrast fibronectin coating enabled 62 (81.6%) of the 76 *S. aureus* isolates to form considerable biofilm masses. However, 18.4% of the *S. aureus*-isolates from RT patients did not grow biofilms neither on uncoated nor on collagen- or fibronectin-coated surfaces.

#### Typing and MSCRAMM-gene detection data

Performing PFGE on the 76 RT patient *S. aureus* isolates identified 65 different PFGE types (figure 1). By setting the cut-off value at 80% for defining unrelated individual strains, 24 different strains resulted from this operation.

**Figure 3:**
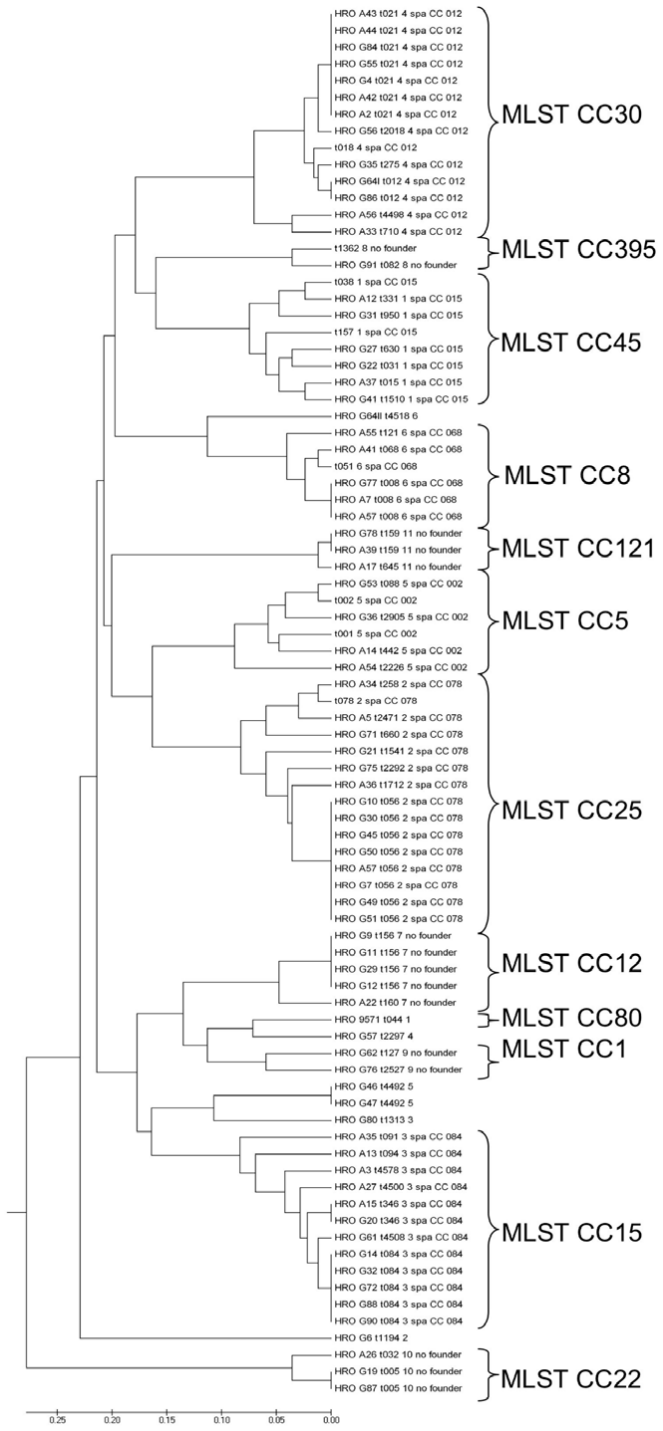
Consensus tree showing the genetic relatedness of the 76 *S. aureus* tonsillar isolates. The 50 *spa*-types were clustered by BURP analysis into 11 clonal complexes. For construction of the consensus tree, several reference strains were included in the BURP clustering. The most common *spa*-type is t056 (n = 8). MLST clonal clusters were deduced from BURP grouping of *spa*-types.

*Spa*-typing of the same *S. aureus* population resulted in 50 different *spa*-types which were clustered by BURP analysis into 11 different clonal complexes (*spa*-/MLST-CC). The constructed consensus tree based upon these *spa*-types (figure 3) is, as expected, different from the consensus tree derived from PFGE-typing. However, there are clear correlations between the PFGE-deduced clusters and the *spa*-/MLST-CCs (figure 1).

The results from the MSCRAMM gene detection by multiplex-PCRs are also listed in figure 1. Of note, each *S. aureus* isolate encoded the *fnbA*, *clfA*, *clfB*, *eno* and *fnbA* genes (data not shown) and a majority carried the *fib* (74%) and *fnbB* (87%) genes. In contrast, only a minority ranging between 30% and 45% of the isolates harbored the *ebpS*, *bbp*, and *cna* genes.

Taking all screened parameters into account, we could distinguish 6 clusters and 22 independent *S. aureus* isolates among the RT patient strains (figure 1).

The first cluster consisted of 14 isolates (A02–G35) belonging to the *spa*-/MLST-CC 30 or 121. Most of the isolates in this cluster were penicillin resistant, positive for *ebpS*, *cna* and *bbp* genes and able to form biofilms only after fibronectin coating.

The second cluster was formed by 10 isolates (G27–G19). In this group the *spa*-/MLST-CCs 45 and 22 were overrepresented. Three common characteristics of this cluster were penicillin resistance, *cna*-gene presence, and biofilm formation on fibronectin-coated surfaces.

The third (G51–G76) and the fourth cluster (G29-G09) formed a supercluster (G29–G76). The third cluster was characterized by *bbp*-gene presence and above average susceptibility to penicillin. The 11 isolates of this cluster predominantly belonged to the *spa*-/MLST-CCs 25 and 1.

Of the 7 isolates of the fourth cluster, some were able to form biofilms not only after fibronectin coating but also on collagen-coated surfaces. Here the *spa*-/MLST-CCs 25 and 12 and penicillin susceptibility were typical characteristics of the isolates.

The fifth cluster, which is also characterized by penicillin susceptibility and biofilm growth only on fibronectin-coated surfaces, has four members (A27–A15). In contrast to the isolates of cluster three, these isolates did not harbor *bbp* and were exclusively members of the *spa*-/MLST-CC 15.

Cluster six consists of 7 isolates (G64I–A55), most of them devoid of *ebps*, *cna* and *bbp*. Frequent *spa*-/MLST-CCs were 30 and 8. The isolates exhibited above-average penicillin susceptibility.

The 21 independent isolates (A13–G91) not associable to one of the described clusters belonged to a wide variety of *spa*-/MLST-CCs (5, 8, 12, 15, 25, 395 and several singletons). They were mostly penicillin-resistant and negative for *ebps*, *cna* and *bbp*. Many of them are poor biofilm formers even on fibronectin-coated surfaces.

Isolate G87 was a single offshoot in the PFGE- consensus tree, but was associated with isolates A26 and G19 in the *spa*-/MLST-CC 22.

#### Immunohistology and FISH

**Figure 4:**
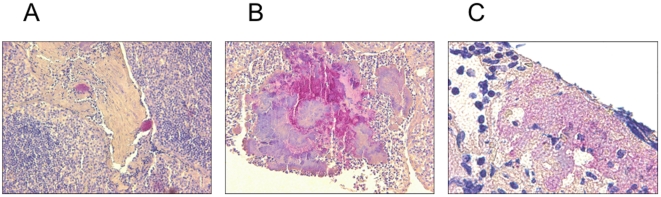
Microscopic aspect of RT-associated tonsillar specimen subjected to immunohistochemistry. *S. aureus*-bacteria were marked by the APAAP staining technique. A: base of a tonsillar crypt (x200) B: necrosis (x200) C: tonsillary epithelium (x1000). These photographs demonstrate that RT-associated *S. aureus* isolates are involved in pathological processes and are not transient colonizers.

For immunohistology and FISH we chose specimen of these 13 patients, where only *S. aureus* and no other bacteria could be grown from the tonsillar tissue. Results from the immunohistologic examination of the tonsil sections from these *S. aureus* positive patients are displayed in figure 4. The microscopic pictures clearly show the association of *S. aureus*-specific staining with histological signs of inflammation like epithelial defects, granular exsudate, necrosis and loose epithelia and thereby demonstrate that *S. aureus* was not a simple transient colonizer at the specific anatomic space.

**Figure 5:**
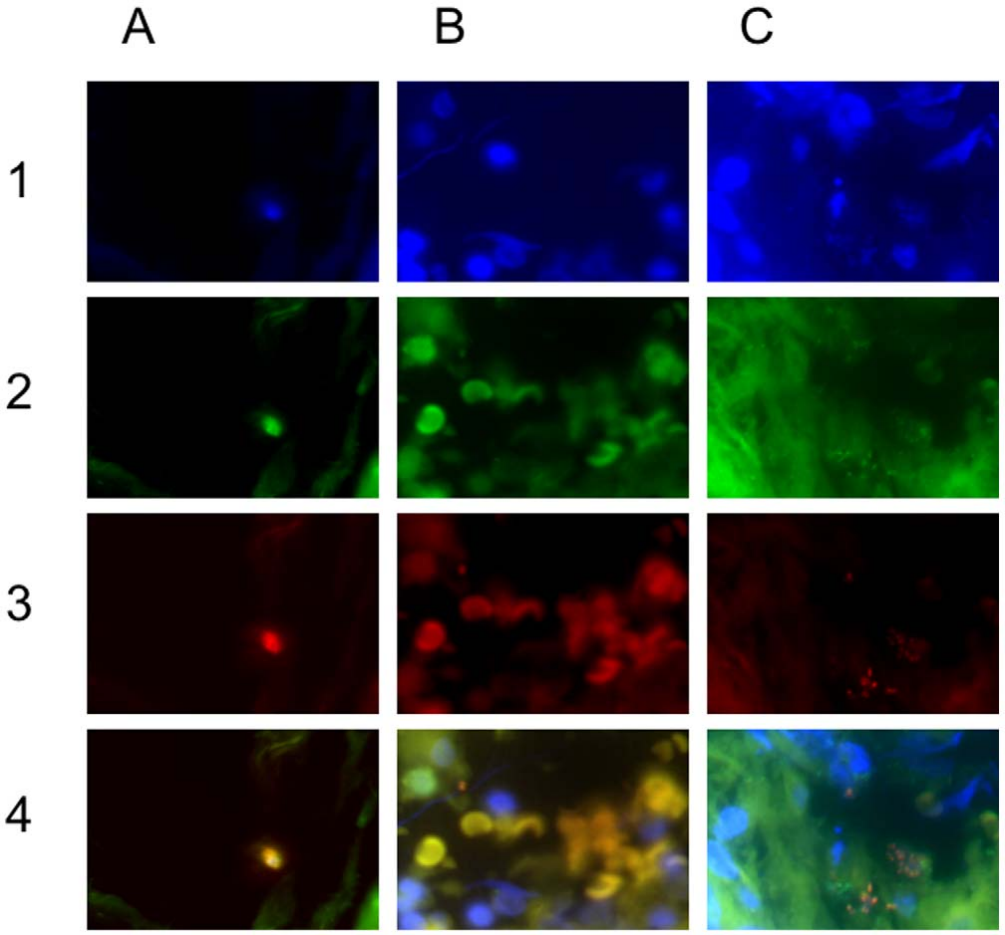
Microscopic aspect of RT-associated tonsillar specimen subjected to fluorescence-in-situ-hybridization. FISH-probes: green (FITC) - eubacteria, red (Cy3) - *S. aureus*, yellow: merge, blue (DAPI): nucleic acids; Column A 1–4: (x1000) cluster of *S. aureus* bacteria wihin a tonsillar crypt. Column B 1–4: (x1000) few *S. aureus* bacteria in the upper left corner of the photograph. Of note, the FISH-probes bound unspecifically to erythrocytes, which in turn miss DAPI-fluorescence (no nuclei). Column C 1–4: (x1000) the proximity of some *S. aureus* bacteria to the DAPI-stained nuclei strongly indicate an intracellular localization. The presence of green-fluorescing non-*S. aureus*-bacteria indicate the status of a polymicrobial infection.

In parallel, FISH performed on paraformaldehyde fixed tonsil sections also demonstrated the presence of *S. aureus*-specific signals (figure 5). For easier comprehension, the fluorescence signal components of the superimposed pictures (line 4) are shown in lines 1 to 3. Thus, the photographs in columns A to C show a cluster of *S. aureus* bacteria within a tonsillar crypt, a small group of *S. aureus* cells in the upper left corner (of note, erythrocytes did not bind the DAPI-stain, but unspecifically the eubacterial and the *S. aureus* probes) and the polymicrobial nature of recurrent tonsillitis, respectively. In column C, *S. aureus* (red to yellow) and other eubacteria (green) can be clearly distinguished.

The proximity of some bacterial signals to signals from eukaryotic nuclei indicated an intracellular localization of such bacteria.

## Discussion

The main objective of the present study was to clarify why RT cases caused by persisting bacterial pathogens often cannot be cured by administration of antibiotics but have to be treated by tonsillectomy. For this purpose, we performed a prospective study with carefully chosen patients suffering from RT or PTA and analyzed not only the presence of microbial pathogens from a variety of patient specimens but also examined crucial molecular data as well as biological functions of the identified bacteria.

Regarding patient numbers, inclusion criteria, and conventional bacteriological analysis the present study is comparable to several studies on RT patients [11]–[14], [16]–[18], [20] and on PTA patients [6], [7], [9] conducted in the last twenty years. However, none of these studies addressed the biological capabilities, i.e. biofilm formation or eukaryotic cell internalization, of the bacteria *in situ* or after isolation. Also, analysis of the clonal diversity of the identified bacteria has not been performed so far.

Consistent with the majority of former studies, *S. aureus* and *Haemophilus* spp. were preferentially associated with RT patients [11], [12], [14], [19]-[21], [43]. Similarly, *S. pyogenes* was the leading single species in material from PTA patients, although much less prominent than previously described [6]-[8]. Comparatively low isolation rates for *S. aureus* and/or high isolation rates for *S. pyogenes* from RT patients as described in some studies [10], [13], [17], [18], [44] could be due to geographical or seasonal effects [25], a selective panel of detection methods, or to temporal variations as described by Timon et al. [20]. In agreement with Lindroos [15], we would state that the impact of *S. pyogenes* in RT pathogenesis has probably been overrated or, alternatively, has decreased in recent years.

A clear advantage of the present study is the inclusion of both children and adults. In contrast to three other studies analyzing both age groups [14], [16], [17], the results were separately evaluated. Noteworthy, children and adults did not differ in the prevalence of single pathogens and to mixtures of pathogens (tables 3 and 4). This indicates that the causative pathogen/-s has/-ve a major impact on pathogenesis and that age-dependent variation of eukaryotic target structures for the identified pathogens is not relevant. Similar to Jeong et al., who analyzed the prevalence of multiple pathogens [14], *S. aureus* and *Haemophilus* spp. were often co-isolated in RT patients, especially in adults. Unlike that study, also the *Streptococcus anginosus* group displayed a high degree of association with *S. aureus* in RT patients. To date, there is not sufficient information available to decide whether these associations rely on symbiotic effects or rather reflect the absence of interference mechanisms similar to those observed between colonizing *S. aureus* and *Streptococcus pneumoniae* strains in the upper respiratory tract [45]-[47].

After establishing *S. aureus* as the predominant pathogenic species associated with RT patients, we asked whether there are RT specific *S. aureus* strains. Utilizing two independent methods of molecular typing, we could show a large genomic diversity among the RT-associated *S. aureus* strains, which was especially remarkable since the patients were recruited from a comparatively small and stable population of about 350.000 inhabitants. Obviously, the capability to cause RT is not confined to a few specifically adapted or equipped *S. aureus* strains.

The next question addresses the potential source of such strains. By comparing the *spa*-derived clonal complex (CC) types of our isolates with those from *S. aureus* strains obtained from healthy nasal carriers in the same geographic region in 2005/06 [48][table 6], we observed a strong overlap in the population structure. This finding suggests that the RT-associated *S. aureus* strains originated from nasal carriage and thus, caused endogenous infections. Consistently, 10 to 35% of asymptomatic adults have been described as persistent nasal *S. aureus* carriers [49] and at least in some settings, pharyngeal carriage rates could be even higher [50].

**Table 6:** Comparison of *spa*-CC–prevalences of *S. aureus* isolates from RT patients and healthy persons with nasal colonization.

*spa*-CC	RT-isolates		nasal isolates [Holtfreter et al. 2007]		
	No. of isolates	%	No. of isolates	%	p-value
CC1	2	2.6	0	0.0	0.1711
CC5	4	5.3	7	6.5	1.0000
CC8	4	5.3	11	10.3	0.2807
CC12	5	6.6	2	1.9	0.1288
CC15	12	15.8	13	12.1	0.5172
CC22	3	3.9	2	1.9	0.6505
CC25	14	18.4	14	13.1	0.4052
CC30	13	17.1	29	27.1	0.1532
CC45	6	7.9	11	10.3	0.7969
CC121	3	3.9	0	0.0	0.0700
CC395	1	1.3	5	4.7	0.4031
singletons	6	7.9	10	9.3	0,7964
excluded	3	3.9	3	2.8	0.6937
total	76	100	107	100	-

Legend: *spa* CC–*spa*-clonal cluster.

After describing the genetic background of the RT-associated *S. aureus* strains, the major issue of this study was addressed–i.e. providing an explanation for the strains' successful persistence in their hosts in spite of repeated antibiotic therapies. Antibiotic cross protection [20], [22] is an improbable mechanism, since the majority of *S. aureus* isolates displayed at least resistance to penicillins and the co-isolated pathogens were less frequently producers of extracellular *β*-lactamases than the *S. aureus* strains (data not shown). Inadequate, since predominantly *S. pyogenes*-directed therapy remains a formal possibility in some cases. Biofilm formation on the tonsillar surfaces cannot be completely excluded as an explanation. At least in specific skin infections, *S. aureus* has been demonstrated in intra-tissue biofilm-like masses [51]. However, the almost complete absence of this capability in our *S. aureus* strains growing on polystyrene or collagen surfaces as well as the absence of material resembling biofilms on selected tonsils as determined by scanning electron microscopy (data not shown) are valid arguments against a leading role of biofilms in RT pathogenesis. Similarly, by using in situ hybridization Swidsinski et al. [32] could demonstrate biofilm-like masses on surgically removed tonsils, but were unable to identify *S. aureus* in these masses.

In contrast, intracellular persistence as the major principle is supported by several experiment-based arguments: first, >90% of *S. aureus* strains were isolated from tonsillar cells (usually white blood cells residing there) by an antibiotic protection assay, >85% of these strains displayed a high degree of internalization in a FACS-based in vitro assay, and the complete subset of strains subjected to FISH and immunoassay-based microscopy of ex vivo material was detected in nucleus-near positions typical for an intracellular status. Consistent with these observations, the molecular analysis confirmed the presence of up to 9 established adhesin genes in the RT-associated *S. aureus* strains, among them the ones encoding the well-known adhesins FnbA and FnbB involved in eukaryotic cell internalization [40], [52]–[54]. However, also all of the in vitro less invasive isolates harbored the *fnbA* gene and varying sets of other adhesin genes, thus not allowing a simple differentiation based on limited genetic analysis.

Which impact has eukaryotic cell internalization on long-term persistence of *S. aureus* in the upper respiratory tract of its human host? In vitro internalization capacity, although on a much lower level, has also been demonstrated for few strains involved in nasal colonization [52]. Additionally, in vitro bacterial transcriptome changes supporting intracellular persistence in respiratory epithelial cells have been studied to great detail [55]. Using ex vivo material from chronic rhino-sinusitis patients, Clement et al. [37] and Plouin-Gaudon et al. [38] convincingly demonstrated intracellular *S. aureus* in 17 out of 27 cases. However, the role of *S. aureus* in chronic rhinosinusitis is not well defined [56]-[58]. Also in our study, the relative contribution of intracellular *S. aureus* to clinical persistence is not completely clear. Additional ex vivo and in vitro comparative analysis of the internalization status of *S. aureus* strains exclusively associated with acute infections could introduce new arguments. Yet, even a high degree of cell invasiveness of such strains would not contradict the importance of that feature for persistence [59].

Which consequences for an alternative antibiotic treatment could be delineated from our results? Contrary to observations on intracellular *S. pyogenes* strains [60], the RT-associated *S. aureus* isolates displayed less often resistance to antibiotics as compared to the PTA-associated strains or those isolated from outpatients of several clinics (table 5). Probably, in *S. aureus* the presence of resistance genes is not coupled to invasin genes as postulated for *S. pyogenes*.

A less frequent occurrence of antibiotic resistance traits could be explained with the protected status of intracellular bacteria, which in turn decreases the selective pressure of antibiotics. Under such circumstances loss of resistance traits should predominantly depend on the duration of the protected status [61], [62]. Yet, the difference between the antibiotic resistance ratios of RT-associated and other *S. aureus* strains was not significant for any tested compounds. Still, every RT-associated isolate was susceptible to clindamycin. This drug has successfully been used for eradication of *S. pyogenes* strains involved in recurrent infections [63], [64] and thus, might as well prove useful to fight intracellular RT-associated *S. aureus* strains. That approach was in fact recommended more than 20 years ago [65] and should now be tested on a larger scale. Alternatively, quinupristin-dalfopristin and oritavancin have been described as active on intracellular *S. aureus* [66]. In fact, selected RT-associated *S. aureus* isolates proved to be fully susceptible to the streptogramin (data not shown), a finding that could extend the therapeutic options. But it should be also taken into consideration that clinical symptoms could also be due to the co-infection with other bactera like *Haemophilus* sp., because in only about one fifth of the RT patients *S. aureus* was the only isolated pathogenic agent.

In conclusion, the present study demonstrates that the majority of RT cases both in children and adults are most probably caused by intracellular persisting *S. aureus* strains and gives experimental data-based indications for an alternative pharmaceutical therapy to avoid surgery and its complications.

## Materials and Methods

### Patients and Statistics

The 254 prospectively recruited participants were juveniles and adults of 1 to 76 years of age (median: 24 years), who were surgically treated at the otolaryngology/head and neck surgery department of the University Hospital Rostock, Germany, from July 2001 until December 2007 because of clinical signs of RT or PTA.

The diagnosis of RT has been based on an anamnestic report of at least three episodes of purulent tonsillar infections and/or odynophagia, tender cervical lymph nodes and pharyngeal pain in the year before tonsillectomy. Criteria for inclusion in the PTA-group were the aspiration of pus and/or persistent pain in the peritonsillar area, trismus and unilateral tonsillar bulge.

General exclusion criteria for both groups were therapy with any antibiotics beside penicillins and cephalosporins within the last two weeks before surgery, severe liver dysfunction, malabsorption, nephrotic syndrome, severe primary and acquired immunodeficiency, hematologic system diseases, pregnancy and lactation.

After tonsillectomy, histological analysis of the specimens from all patients was routinely performed to verify the clinical diagnosis.

This study was conducted following the approval of the ethics commission of the Medical Association Mecklenburg-Vorpommern (Reg.-no. II HV 17/2001). We got written informed consent from all participants, which were involved in our study.

### Culture of Extra- and Intracellular Bacteria (Antibiotic Protection Assay)

**Figure 6:**
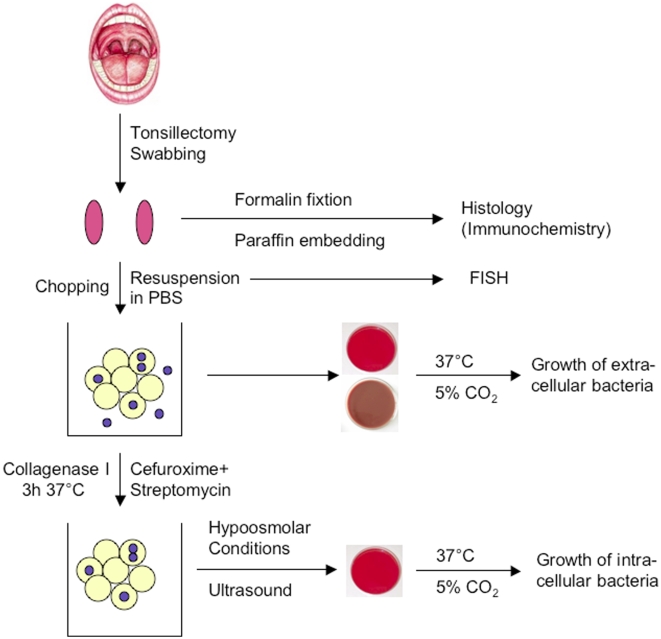
Flow chart illustrating the processing of tonsillar specimens as described in the “Materials and Methods”-section.

Immediately prior to surgery the tonsils were swabbed using sterile wooden spatula (figure 6). The swabbed material was suspended in 10 ml phosphate buffered saline (PBS, pH 7.4) and kept at 4°C. The surgically removed tonsils were transferred to 10 ml PBS and also kept at 4°C. All specimens were transported in cooled containers and processed within two hours after sampling.

Suspended tonsillar cells from the swabs were centrifuged for 10 min at 800×g and 4°C. After discarding the supernatant, cells were resuspended in 1 ml fresh PBS. For subsequent FISH analysis about 3×30 µl of this cell suspension was streaked onto three microscope slides.

In addition, 10 µl aliquots were used to inoculate sheep blood and chocolate agar plates. The culture media were incubated over night at 37°C under a 20% O_2_/5% CO_2_ atmosphere. The remaining suspension was supplemented with streptomycin and cefuroxime, both to final concentrations of 100 µg/ml, and incubated for 3 h at 37°C under ambient air to kill extracellular bacteria while not affecting intracellular bacteria ([36], [67], see also below for control experiments).

Upon arrival in the laboratory, surgical tonsil specimens were cut in small pieces using sterile instruments. About half of the tissue was fixed in formalin, dehydrated an embedded into a paraffin matrix for histology. The remaining tonsil tissue pieces were immersed in 1 ml PBS and vortexed. Ten µl aliquots from the crude suspension were plated and cultured as described for the untreated cell suspensions. To achieve a homogenous cell suspension and best possible access for antibiotic supplements, the remaining crude suspensions were supplemented with collagenase I-solution (Sigma) to a final concentration of 100 mg/ml (12500 collagen digestion units/ml) and the above described antibiotics, incubated and further processed like the swab-derived cell suspensions.

After 3 hrs of incubation the cells were disrupted by exposure to ultrasound (Bandelin Electronic UW2200) and subsequent hypoosmolar conditions. The lysates were cultured on solid media like the untreated cell suspensions.

To verify the thorough killing of extracellular bacteria under the conditions chosen for the antibiotic protection assay, varying numbers of *S. aureus* ATCC 25923 were added to subconfluent HEp-2 cell monolayers (multiplicity of infection, MOI: 0.01-100) and allowed to internalize for periods between one to four hours. Antibiotics were added at above described concentrations after 0 to 120 minutes to the co-cultures and allowed to react for periods up to four hours. The eukaryotic host cells were mechanically suspended and bacteria were quantified with respect to their extra- or intracellular location. At antibiotic exposure periods of three hours consistently no extracellular bacteria could be detected, while the amount of intracellular bacteria increased with respect to the initial inoculum and the coincubation period.

As an additional control suspensions from three tonsil preparations were spiked with 100 µl of *S. aureus* ATCC 25923 suspension containing 0.5×10^4^ CFU/ml and processed as above. While the inoculated bacteria could be detected in absence of antibiotics, no *S. aureus* was cultured from an extracellular location after the full exposure period to the antibiotics.

### Bacteriological Identification and Susceptibility Testing

Identification of bacterial species was performed using established methods of the accredited diagnostic laboratory. All pathogenic strains were stored at –80°C in microbank tubes (Pro-lab Diagnostics) utilizing isolates from the first laboratory passage.

*S. aureus* was identified by the Pastorex Staph-Plus latex agglutination kit (BIO-RAD). Sero-types of β-hemolysing streptococci were determined by the Pastorex Strep latex agglutination test (BIO-RAD). The species identification of such streptococci was achieved by 16S rRNA gene sequence analysis. Other bacterial species were differentiated using the Vitek 2 device or appropriate API-systems (bioMérieux).

Antibiotic resistance tests of *S. aureus* isolates were performed with penicillin (10 µg), oxacillin (1 µg), cefuroxime (30 µg), levofloxacin (5 µg), gentamycin (10 µg), streptomycin (10 µg), erythromycin (15 µg), teicoplanin (30 µg), vancomycin (30 µg) and cotrimoxacole (1,25/23,75 µg) using a disc diffusion method on Mueller-Hinton agar (Oxoid) according to the CLSI (Clinical and Laboratory Standards Institute) directives (M100-S17M2-A9). MICs of moxifloxacin and clindamycin were determined by the E-test method (AB BIODISK).

### Pulsed-Field Gel Electrophoresis

Molecular typing to demonstrate the epidemiological relation of the isolated *S. aureus* strains was performed by pulsed-field gel electrophoresis (PFGE) of chromosomal DNA digested with the endonuclease SmaI (Roche) by using a CHEF-DR III Pulsed Field Electrophoresis System (BIO-RAD) according to established protocols [68] with minor modifications using following settings - initial switch time: 5 sec., final switch time: 35 sec., voltage: 5.5 V/cm, included angle: 120° run time: 22 h.

The chromosomal DNA restriction patterns were interpreted with help of the GelCompar™-Software (version 4.1, Applied Math) using the UPGMA algorithm with Dice coefficients. According to this program and the criteria of Tenover et al. [69], strains with PFGE pattern relatedness below 80% were regarded as different clones.

### DNA Extraction, *Spa*-Typing and MSCRAMM-Multiplex-PCR

Bacterial genomic DNA was purified using QIAamp DNA Mini Kit (Qiagen). *Spa*-typing was performed by amplification and sequencing of the variable X region of the *spa*-gene. Obtained forward and reverse sequence chromatograms were analyzed using the Ridom StaphType software (http://spaserver.ridom.de/, Ridom) [70]. *Spa*-types were clustered into different groups by the BURP algorithm.

The presence of MSCRAMM-genes in the *S. aureus* isolates was monitored by a multiplex PCR as described by Tristan et al. [39]. Two primer sets (PCR1: *bbp*, *cna*, *ebpS* and *eno*; PCR2: *fnbA*, *fnbB*, *fib*, *clfA* and *clfB*) were used for multiplex PCR. The thermal cycling conditions included preincubation at 94°C for 5 min followed by 25 cycles of denaturation at 94°C for 1 min, annealing at 55°C for 1 min and extension at 72°C for 1 min, with a final extension of 72°C for 10 min. PCR products were analyzed on 1% agarose gels and stained with ethidium bromide.

### Flow Cytometric Internalization Assay

A well-established and widely used FACS-based internalization assay was performed as described previously [71] using a FACSCalibur (Becton Dickinson) and the following modifications. Subconfluent monolayers of human A549 lung carcinoma cells (DSMZ) grown in DMEM/10% calf serum were inoculated with the respective 5-(6)-caboxyfluoresceinsuccinimidylester stained (CFSE; Sigma-Aldrich) *S. aureus*-strain at a multiplicity of infection (MOI) of 30. The fluorescence signal of adherent but not internalized bacteria was quenched by adding 0.2% trypan blue (Sigma-Aldrich) to each sample before measurement. The non-invasive *S. carnosus* strain TM300 (ATCC 51365) was used as negative control to define the background of the fluorescence signal. *S. aureus* strains that showed less than 20% invasiveness comparing to the reference strain Cowan I (ATCC 12598) were defined as non-invasive [52], [53].

### Biofilm Analysis

Assays to quantify staphylococcal biofilm formation while maintaining the bacteria in trypticase soy broth (TSB) are widely established [72], [73]. The biofilm experiments were performed in 96-well polysterene microtiter plates (Greiner). The well surfaces were used either uncoated or coated with human fibronectin (Roche) or human collagen type I (Biomol). Coating was performed overnight at 4°C with the matrix molecules at concentrations of 50 µg/ml. Unbound matrix molecules were removed by three washes with PBS.

Precultures were obtained by direct inoculation of the tonsillar *S. aureus* isolates into a 1 ml trypticase soy broth (TSB, Oxoid) and incubation overnight at 37°C. Then the bacteria were harvested by centrifugation, resuspended in TSB to an optical density (OD 600 nm) of 0.5 and 100 µl of this suspension containing 10^4^ CFU/ml were inoculated into each well. Planktonic bacteria were removed by aspiration of the medium after 24 h or 48 h incubation and biofilms were stained with 0.1% safranin after washing twice with PBS. The OD 492 nm of the stained biofilms was determined according to Lembke et al. [74].

Analyses were performed on three independent occasions and two technical replicates with each isolate. Results are shown as mean values of all experiments. The excellent biofilm forming *S. aureus* strain ATCC 25923 [75] and the biofilm negative *S. carnosus* strain TM300 (ATCC 51365) [76] were used as references.

### Immunohistochemistry

Immunohistochemical assays were performed on formalin-fixed, paraffin-embedded, then deparaffinized and rehydrated 1 µm sections of the tonsillar tissue samples as described by Guarner et al. [77] using the polyclonal rabbit anti-*S. aureus* antibody (BIODESIGN International) as primary antibody and the APAAP staining technique (DAKO Cytomation).

### Fluorescence *In Situ* Hybridization (FISH)

FISH was carried out as described by Krimmer et al. [78] with minor modifications. Briefly, for deparaffination the tissue sections were treated twice with xylene and then with xylene-ethanol (1∶1). For permeabilization, the bacteria were treated with 200 mg/l lysostaphin and 12.5 mg/l lysozyme in PBS/0.05% Triton-X-100. *In situ* hybridization of bacteria was performed using the following oligonucleotides: EUB-338 5′-GCT-GCC-TCC-CGT-AGG-AGT-3′ FITC-labeled (eubacterial probe; [79]) and Sau-69 5′-GAA-GCA-AGC-TTC-TCG-TCC-G-3′ Cy3-labeled (*S. aureus*–probe; [80]). Both probes were synthesized by Eurogentec. Vectashield® Mounting Medium with DAPI (Vector laboratories) was used on hybridized slides. Finally, the slides were analyzed with a BX60 fluorescence microscope (Olympus).

## Supporting Information

Abstract S1 Abstract in German. Translation of the Abstract into German
(0.03 MB DOC)
