# Supplementary material for: Intracellular Persisting Staphylococcus aureus Is the Major Pathogen in Recurrent Tonsillitis
Source: PLoS One. 2010 Mar 1;5(3):e9452. doi: 10.1371/journal.pone.0009452 (PMC2830486; doi:10.1371/journal.pone.0009452)
Supplement: Abstract S1 — Abstract in German. Translation of the Abstract into German (0.03 MB DOC) [file pone.0009452.s001.doc]

## Intrazellulär persistierende *Staphylococcus aureus*–Stämme sind das vorherrschende Pathogen bei chronisch rezidivierender Tonsillitis

Andreas E. Zautner^1*^, Merit Krause^2^, Gerhard Stropahl^3^, Silva Holtfreter^4^, Hagen Frickmann^1^, Claudia Maletzki^1^, Bernd Kreikemeyer^1^, Hans Wilhelm Pau^2^, Andreas Podbielski^1^

^1^Institut für Medizinische Mikrobiologie, Virologie und Hygiene, Rostock, Deutschland

^2^Klinik und Poliklinik für Hals-Nasen-Ohrenheilkunde, Kopf- und Halschirurgie „Otto Körner", Rostock, Deutschland

^3^Institut für Pathologie, Universitätsklinikum Rostock, Deutschland,

^4^Institut für Immunologie und Transfusionsmedizin, Ernst-Moritz-Arndt Universität, Greifswald, Deutschland

**Zusammenfassung**

***Hintergrund*** Die beiden wichtigsten Indikationen für eine Tonsillektomie sind die rezidivierende Tonsillitis (RT) und der Peritonsillarabszess (PTA). Im Gegensatz zu PTA, die in erster Linie operativ behandelt werden, wird die RT oftmals erst nach einer Reihe von gescheiterten, konservativ-medikamentösen Behandlungsversuchen mittels Tonsillektomie therapiert. Obwohl die bakteriologischen Ursachen der RT bereits mehrfach untersucht worden, sind die Gründe für den fehlenden Erfolg konservativer Therapieansätze weiterhin nicht geklärt.

***Methoden*** Im Rahmen einer prospektiven Studie wurde das Gewebe ektomierter Tonsillen von 130 RT-Patienten sowie von 124 PTA-Patienten auf das Vorhandensein extra- und intrazellulärer Bakterien mit Hilfe eines Antibiotika-Protektionsassays untersucht. Die *Staphylococcus aureus*-Isolate der RT-Patienten wurden mittels Pulsfeldgelelektrophorese (PFGE), *spa*-Typisierung und MSCRAMM-Gen-PCR charakterisiert. Ihre Fähigkeit zur Biofilm-Bildung wurde untersucht und ihre Zellinvasivität mit einem durchflusszytometrischen Invasionsassay (FACS), Fluoreszenz-*in situ*-Hybridisierung (FISH) und Immunhistologie nachgewiesen.

***Ergebnisse*** Bei den RT-Patienten war mit 57,7% *S. aureus* die am häufigsten isolierte Bakterienart, während *Streptococcus pyogenes* mit 20,2% bei den PTA-Patienten die vorherrschende Spezies darstellte. Mit Hilfe drei verschiedener Methoden (FACS, FISH, Antibiotika-Protektionsassay) konnte demonstriert werden, dass sich nahezu alle RT-assoziierten *S. aureus*-Stämme intrazellulär befanden. Die Ergebnisse der MSCRAMM-Gen-PCR bestätigten, dass 87% dieser *S. aureus*-Isolate zu invasiven Stämmen gehörten und nicht nur apathogene Kolonisatoren waren. Basierend auf den PFGE-Analysen und der *spa*-Typisierung gehört der überwiegende Teil der *S. aureus*-Isolate verschiedenen klonalen Linien an.

***Schlussfolgerungen*** Unsere Ergebnisse demonstrieren, dass intrazellulär persistierende *S. aureus*-Stämme die häufigste Ursache der RT sind, und zeigen, dass *S. aureus* diese Lokalisation nutzt, um sich den Einflüssen des Wirts-Immunsystems und der gegebenenfalls verabreichten Antibiotika zu entziehen.
